# Impact of vaccination on pertussis-related hospital admissions in children in Scotland from January 2013 to July 2024: a cohort study

**DOI:** 10.2807/1560-7917.ES.2025.30.39.2500270

**Published:** 2025-10-02

**Authors:** Taimoor Hasan, Ewan Wilkinson, Valérie Decraene, Ariadni Kouzeli, Cheryl Gibbons, Vera Chua, Roberto Vivancos, Sam Ghebrehewet

**Affiliations:** 1 Public Health Scotland, Edinburgh, United Kingdom; 2 Institute of Medicine, University of Chester, Chester, United Kingdom; 3 UK Health Security Agency, London, United Kingdom; 4 NIHR Health Protection Research Unit in Gastrointestinal Infections, Norwich, United Kingdom; 5 Warwick Medical School, University of Warwick, Coventry, United Kingdom; 6 NIHR Health Protection Research Unit in Emerging and Zoonotic Infections, Liverpool, United Kingdom

**Keywords:** Childhood immunisation, health inequalities, vaccine preventable disease, deprivation, vaccine effectiveness, pertussis

## Abstract

**BACKGROUND:**

In Scotland, the number of pertussis infections recorded in children in 2024 was the highest of any year in the last decade. The protective role of vaccination against severe infection and associated hospitalisations has not been assessed.

**AIM:**

To investigate the effect of vaccination and sociodemographic factors on pertussis-related hospitalisations in Scottish children aged under 18 years.

**METHODS:**

In a retrospective cohort study, laboratory-confirmed pertussis cases from January 2013 to July 2024 were extracted from the national electronic surveillance system and linked to hospitalisation data from Scottish Morbidity Records and vaccination data from the national immunisations database. The outcome was a pertussis-associated hospitalisation. Multivariable logistic regression was used to calculate odds ratios (OR) for the association between vaccination status and hospitalisation, adjusted for age, sex, ethnicity and deprivation status.

**RESULTS:**

There were 3,982 laboratory-confirmed cases of pertussis during the study period. Children fully vaccinated for age had significantly lower odds of hospitalisations than unvaccinated children (adjusted OR (aOR): 0.31; 95% CI: 0.21–0.46). Being partially vaccinated for age did not significantly reduce hospitalisations relative to unvaccinated children (aOR: 0.80; 95% CI: 0.47–1.33). In the univariable analysis, children living in the most deprived areas had significantly more hospitalisations than those in the least deprived areas (OR: 3.90; 95% CI: 2.41–6.56). This association was not significant when adjusted for the effect of vaccination (aOR: 1.47; 95% CI: 0.84–2.66).

**CONCLUSIONS:**

Fully vaccinated children had significantly lower odds of hospitalisation, indicative of less severe disease. This emphasises the importance of fully vaccinating children according to the childhood immunisation schedule.

**Key public health message**

**What did you want to address in this study and why?**

Pertussis, or whooping cough, is a highly infectious disease caused by the bacterium *Bordetella pertussis*. It can cause serious illness to infants and young children and can lead to hospitalisation and death. We wanted to examine whether childhood pertussis vaccination helps protect children aged under 18 years in Scotland from becoming seriously ill and needing hospital care.

**What have we learnt from this study?**

Children fully vaccinated according to the vaccination schedule were less likely to be hospitalised with a pertussis infection. Our study shows that the risk of hospitalisation was reduced by 69% in fully vaccinated children aged 8 weeks to 17 years. Being fully vaccinated also reduced the risk of pertussis hospital stays in children aged 8 weeks to 1 year by 88%.

**What are the implications of your findings for public health?**

The impact of childhood pertussis vaccination in preventing pertussis-related hospitalisations highlights the importance of vaccination programmes in reducing the load on healthcare systems. To achieve the desired public health outcomes, it is vital to make vaccines easy to access, ensure they are administered on time, and to remove any obstacles to vaccination, such as vaccine hesitancy and misinformation.

## Introduction

Pertussis, or whooping cough, is a highly infectious disease caused by the bacterium *Bordetella pertussis* [1]. Pertussis can be particularly serious for infants and young children and may result in hospitalisation and even death [2-4]. In the United Kingdom, pertussis immunisation has been part of the primary childhood immunisation programme since 2003 [1]. Infants currently receive a single dose of a pertussis-containing vaccine at 8, 12 and 16 weeks of age, as well as a pre-school booster at 3 years and 4 months of age [5]. Due to a rise in pertussis cases in 2012 in infants younger than 3 months, a maternal vaccination programme was implemented [1]. Since 2012, pregnant women are offered a pertussis-containing vaccine between 16 and 32 weeks of gestation to provide transplacental passive immunity to newborns and to protect them in the first few weeks of life, before them becoming eligible for their primary vaccinations [1]. Current uptake in Scotland of the primary three doses of pertussis-containing 6-in-1 vaccine in children by 12 months of age is 94.4%, and of the pre-school booster by 5 years of age is 89.3% [6]. The uptake of maternal pertussis vaccination in Scotland from April 2023 to March 2024 was 82.3% [7].

Pertussis case numbers have generally stayed at a lower level in the UK (444 and 752 cases of all ages in Scotland in 2018 and 2019, respectively) given the success of childhood and maternal immunisation programmes [7,8]. During the COVID-19 pandemic, pertussis cases were very low (< 5 cases in 2021 and 2022) because of health protection restrictions in place [7]. However, in 2023–24, the years following the pandemic, there has been a global surge in the number of pertussis cases. In the first half of 2024, 4,849 cases were reported in Scotland [7]. In Europe, in the first quarter of 2024, more than 32,000 cases were reported [9]. This observed rise in pertussis infections has been attributed to multiple factors, including expected epidemic peaks every 3–5 years because of a cyclical pattern of pertussis infections, the presence of individuals who are unvaccinated or whose vaccinations are not current, waning immunity and decreased natural boosting of immunity in the overall population as a result of decreased circulation during the COVID-19 pandemic, causing an increased pool of susceptible individuals [9].

Pertussis infection is usually more severe in infants, particularly in those under 3 months of age that are not yet protected by primary vaccinations [2,3]. A pre-pandemic study from England showed an association between prolonged pertussis-related hospital stays for infants with factors including prematurity, young age, additional respiratory illness and lack of maternal vaccination during pregnancy [4]. Evidence from Italy and Sweden has shown that young age and being unvaccinated is significantly associated with increased hospitalisations and increased severity of disease with pertussis [10,11].

In Scotland, the magnitude of the protective effect of vaccination against hospitalisations from severe pertussis infection in children has not been evaluated. In light of the recent rise in pertussis cases in 2024, understanding the impact of the vaccination programme can help target efforts to improve uptake. This study aims to evaluate the impact of childhood pertussis vaccination in Scotland by examining the association between vaccination status and pertussis-related hospital admissions in children aged 17 years and under.

## Methods

### Study design and population 

We undertook a retrospective cohort study. The study population included individuals aged 17 years and under with laboratory-confirmed pertussis in Scotland from 1 January 2013 to 31 July 2024. 

### Study setting

Scotland is part of the UK and has its own autonomous National Health Service (NHS). Public Health Scotland (PHS) is the national public health body and is part of NHS Scotland. 

Pertussis is a legally notifiable disease in Scotland [12]. When a pertussis case is confirmed at a local NHS diagnostic laboratory, it is added to the Electronic Communication of Surveillance in Scotland (ECOSS) system. All childhood vaccinations in Scotland are recorded when they are administered and compiled in the Scottish Immunisation Recall System (SIRS). Information on hospital admissions in Scotland are recorded in the Scottish Morbidity Record 01 (SMR01) dataset.

### Exposure

The exposure of interest in this study was the vaccination status of laboratory-confirmed pertussis cases. Data on pertussis-containing vaccine administration were extracted from SIRS up to 31 July 2024, as they were the latest data available at the time of extraction in August 2024. Vaccination status was defined based on whether a child was up to date with the recommended number of doses for their age according to the UK routine childhood immunisation schedule at the date of pertussis infection [5]. The three categories were: (i) fully vaccinated for age, (ii) partially vaccinated for age and (iii) unvaccinated.

### Outcome

The study outcome was a hospital admission with a pertussis infection within 30 days of laboratory confirmation of the infection. Episode-level data with an International Classification of Diseases 10^th^ revision (ICD-10) codes [13] for pertussis (A37.0, A37.9) within any of the diagnosed conditions were extracted from SMR01 from 1 January 2013 to 30 August 2024. SMR01 episodes were then aggregated to hospital stay level for individuals based on the Community Health Index (CHI) number, the unique patient identifier, counting multiple episodes of pertussis once within a single hospital stay. 

### Covariates

To assess the association between patient characteristics and pertussis-associated hospital admissions, covariates considered in the study included age, sex, ethnicity, deprivation status and comorbidities.

Data were deterministically linked across all sources using the CHI number. Scottish Index of Multiple Deprivation (SIMD) was used as a proxy for deprivation status and was determined for each case using the postcode information available in SMR01. Ethnicity information at individual level was obtained from Public Health Scotland’s internal dataset. Ethnicity was categorised based on ethnic group classification in the Scotland Census, where each individual is classified according to their own perceived ethnic group and cultural background (White, Mixed or multiple ethnic groups, Asian, Scottish Asian or British Asian, African, Scottish African or British African, Caribbean or Black, other ethnic groups) [14]. 

Comorbidities were measured using the Charlson comorbidity index [15]. For individuals with a pertussis-associated hospital admission stay, ICD-10 codes for that stay were used to calculate the Charlson comorbidity score. For all other individuals, SMR01 records were searched for 10 years preceding the date of infection to find their most recent hospital admission to calculate the comorbidity score. Children who did not have any hospital admission in this period were classified as having no comorbidities.

### Statistical analysis

Children under 8 weeks old at the time of infection were excluded from the primary analysis. Additionally, all categories of ethnicity other than White, Asian, South Asian or British Asian and Mixed or multiple ethnicities were condensed into one category ‘Other’ to preserve statistical power, on account of small numbers.

We assessed the univariable association of hospitalisations with vaccination status and other covariates by chi-square tests and simple logistic regression. To assess the association between vaccination status and pertussis-associated hospital admissions adjusted for age, sex, deprivation and ethnicity, we developed a multivariable logistic regression model with a binary outcome (hospital admission or no admission). The covariates controlled for in the model were identified a priori through literature searches, and were included in the model as categorical variables. The multivariable analysis was undertaken on the complete dataset; missing data for any of the covariates were retained in the model by creating an additional category for that variable labelled as ‘missing data’. We only considered imputing missing values for a variable when percentage missingness was above the threshold of 10%. This decision was based on recommendations from statistical literature that when missingness is below 10%, imputing is unlikely to provide any added value to the results [12,16]. Children who were aged under 8 weeks old at the time of their infection were excluded from the model due to lack of complete maternal pertussis vaccination data at the time of analysis.

To estimate any differential association between a covariate and hospitalisation by vaccination status, models were fitted after including an interaction term between the covariates and the exposure variable. Interaction terms were only retained in the final model if found to be significant at the 95% confidence level through a likelihood ratio test. Additionally, to assess any significant difference in vaccine effectiveness against hospitalisation by period of infection, we ran logistic regression models separately on pertussis cases that occurred in 2024 and those that occurred from 2013 to 2023. A logistic regression model was selected over a model type that accounts for follow-up time because the use of the outcome as ‘hospital admission within 30 days of the laboratory confirmation of infection’ meant that there was effectively a fixed follow-up period for outcome ascertainment. P values of < 0.05 were considered significant. The fit of the model was assessed using measures of accuracy, sensitivity and specificity. Statistical analyses were carried out using R (version R-4.1.2).

### Subgroup analysis

To further investigate the evidence of a greater adverse impact of pertussis on children aged under 1 year, multivariable logistic regression analysis was undertaken to assess the association between vaccination status and hospital admission for this group, adjusted for the effect of sex, ethnicity and deprivation status. Children aged under 8 weeks were also excluded from this analysis.

## Results

There were 3,982 laboratory-confirmed cases of pertussis in children aged 17 years and under in Scotland between 1 January 2013 to 31 July 2024 (Table 1). Of those, 264 (6.6%) cases had a pertussis-associated hospital stay. None of these children had more than one hospital stay. More than half of the cases occurred in 2024 (Figure). A breakdown of cases occurring in 2024 and those that occurred earlier by demographic characteristics and hospitalisation status is provided in Supplementary Table S1.

**Table 1:** Characteristics of the study population of children aged ≤ 17 years with laboratory-confirmed pertussis, Scotland, 1 January 2013–31 July 2024 (n = 3,982)

Characteristics	Total	Hospitalised cases		Non-hospitalised cases		p value
		n	%	n	%	
Case numbers	3,982	264	6.6	3,718	93.4	
Age groups						
0–8 weeks	74	58	78.4	16	21.6	< 0.05^a^
8 weeks–** < **1 year	300	128	42.7	172	57.3	
1–3.5 years	412	31	7.5	381	92.5	
3.6–9 years	1,030	19	1.8	1,011	98.2	
10–12 years	977	14	1.4	963	98.6	
13–17 years	1,189	14	1.2	1,175	98.8	
Sex						
Females	2,059	134	6.5	1,925	93.5	0.84^a^
Males	1,923	130	6.8	1,793	93.2	
Ethnicity^b^						
White	3,594	235	6.5	3,359	93.5	0.07^c^
Asian	85	12	14.1	73	85.9	
Black	4	1	NA	3	NA	
Mixed	77	4	5.2	73	94.8	
African	22	1	NA	21	NA	
Other	41	6	NA	35	NA	
Missing data	159	5	3.1	154	96.9	
Scottish Indices Multiple Deprivation (quintiles)						
1 (most deprived)	700	84	12	616	88	< 0.05^a^
2	637	47	7.4	590	92.6	
3	681	37	5.4	644	94.6	
4	749	35	4.7	714	95.3	
5 (least deprived)	889	25	2.8	864	97.2	
Missing data	326	36	11	290	89	
Vaccination status						
Fully vaccinated for age	3,162	84	2.7	3,078	97.3	< 0.05^a^
Partially vaccinated for age	322	36	11.2	286	88.8	
Unvaccinated	498	144	28.9	354	71.1	
Comorbidities**^d^**						
No comorbidity	3,800	254	6.7	3,546	93.3	0.63^a^
1–2 comorbidities	182	10	5.5	172	94.5	

^a^ The p values are based on Chi-square tests. Statistical significance is achieved at p value < 0.05.

^b^ Ethnicity was categorised based on ethnic group classification in the Scotland Census, where each individual is classified according to their own perceived ethnic group and cultural background [14].

^c^ The p value is based on Fisher’s exact test.

^d^ Measured by the Charlson comorbidity score [15].

Percentages are presented by rows.

**Figure:**
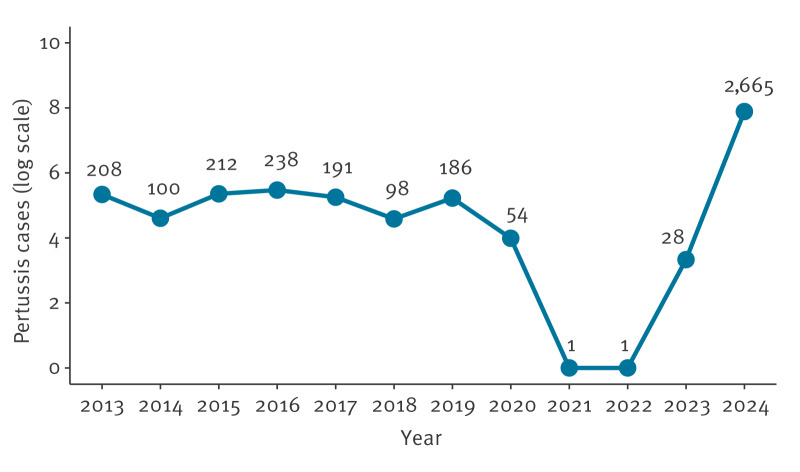
Number of laboratory-confirmed pertussis cases by year in children aged ≤ 17 years, Scotland, 1 January 2013–31 July 2024 (n = 3,982)

### Primary analysis

In the multivariable analysis, children who were fully vaccinated for their age had significantly lower odds of hospital admission with a pertussis infection compared with children who were unvaccinated, adjusted for the effect of age, sex, ethnicity and deprivation (adjusted odds ratio (aOR): 0.31; 95% CI: 0.21–0.46) (Table 2). Being partially vaccinated against pertussis showed a significant protective effect against hospital admission in the univariable model compared with the unvaccinated children (odds ratio (OR): 0.47; 95% CI: 0.30–0.69). However, this was not statistically significant (aOR: 0.80; 95% CI: 0.47–1.33) in the multivariable model.

**Table 2:** Association between vaccination status and pertussis-associated hospital admissions in children aged ≤ 17 years^a^, Scotland, 1 January 2013–31 July 2024 (n =  3,908)

Characteristics^a^	Unadjusted OR	95% CI	Adjusted OR	95% CI^b^
Vaccination status				
Unvaccinated	Reference		Reference	
Fully vaccinated for age	0.09	0.07–0.13**	0.31	0.21–0.46**
Partially vaccinated for age	0.47	0.30–0.69*	0.80	0.47–1.33
Age				
8 weeks–** < **1 year	Reference		Reference	
1–3.5 years	0.11	0.07–0.17**	0.19	0.11–0.30**
3.6–9 years	0.02	0.01–0.03**	0.04	0.02–0.07**
10–12 years	0.02	0.01–0.03**	0.04	0.02–0.07**
13–17 years	0.03	0.01–0.04**	0.03	0.01–0.05**
Sex				
Female	Reference		Reference	
Male	1.00	0.76–1.33	0.80	0.57–1.10
Ethnicity				
White	Reference		Reference	
Asian	1.98	0.87–3.93	2.33	0.86–5.67
Other^c^	1.12	0.50–2.18	0.80	0.27–2.08
Missing data	0.47	0.14–1.13	0.74	0.21–2.02
Deprivation (SIMD quintiles)				
1 (most deprived)	3.90	2.41–6.56*	1.47	0.84–2.66
2	2.53	1.50–4.40*	1.31	0.72–2.45
3	1.70	0.97–3.03	1.25	0.66–2.39
4	1.54	0.87–2.73	1.23	0.65–2.34
5 (least deprived)	Reference		Reference	
Missing data	3.79	2.14–6.79	2.31	1.18–4.54

CI: confidence interval; OR: odds ratio; SIMD: Scottish Index of Multiple Deprivation.

^a^ Children under 8 weeks old were not included in the analysis because data on maternal pertussis vaccination status were unavailable.

^b^ Adjusted for age, sex, ethnicity and deprivation.

^c^ African, Scottish African or British African, Caribbean or Black and other ethnic groups grouped into one category.

Statistical significance is indicated as ** p value < 0.01 and *p value < 0.05.

Age was independently associated with hospital admissions, with children aged under 1 year at significantly higher odds of admissions than older children (1–17 years). Based on the univariable model, children living in the bottom two most deprived SIMD quintiles had 3.90 and 2.53 times higher odds, respectively, of being admitted to the hospital compared with the children living in the least deprived quintile. However, this association was not significant when adjusted for the effect of other independent variables in the multivariable model (quintile 1 = aOR: 1.47; 95% CI: 0.84–2.66; quintile 2 = aOR: 1.31; 95% CI: 0.72–2.45). Asian children had higher odds of pertussis-associated hospital admission compared with White children in the multivariable model, but the association was not significant at the 95% confidence level (aOR: 2.33; 95% CI: 0.86–5.67).

There were no statistically significant interactions between covariates and the exposure variable (vaccination status). Additionally, there was no statistically significant difference in the effectiveness of vaccine against pertussis-associated hospital admissions in cases that occurred in 2024 and those that occurred between 2013 and 2023. Separate multivariable logistic regression model outputs on association between vaccination status and hospital admissions for pertussis cases from 2013 to 2023 (n = 1,317) and for cases in 2024 (n = 2,665, up to 31 July) are presented in Supplementary Table S2.

### Subset analysis

Among cases aged under 1 year excluding those under 8 weeks, children who were fully vaccinated for age had significantly lower odds to be admitted to the hospital with pertussis infection compared with unvaccinated children (aOR: 0.12; 95% CI: 0.06–0.22) (Table 3). None of the other covariates had a statistically significant association with the outcome.

**Table 3:** Association between vaccination status and pertussis-associated hospital admissions in children aged under 1 year^a^, Scotland, 1 January 2013–31 July 2024 (n = 300)

Characteristics^a^	Adjusted OR	95% CI^b^	p value
Vaccination status			
Unvaccinated	Reference		Reference
Fully vaccinated for age	0.12	0.06–0.22	< 0.01
Partially vaccinated for age	0.63	0.33–1.22	0.17
Sex			
Female	Reference		Reference
Male	0.80	0.47–1.35	0.41
Ethnicity			
White	Reference		Reference
Asian	2.57	0.53–15.18	0.26
Other^c^	0.55	0.17–1.61	0.28
Missing data	0.48	0.02–5.73	0.57
Deprivation (SIMD quintiles)			
1 (most deprived)	1.17	0.45–3.13	0.75
2	1.27	0.46–3.57	0.65
3	1.34	0.45–4.08	0.60
4	0.55	0.17–1.81	0.33
5 (least deprived)	Reference		Reference
Missing data	1.54	0.49–5.01	0.47

CI: confidence interval; OR: odds ratio; SIMD: Scottish Index of Multiple Deprivation.

Statistical significance is at the 95% (p value < 0.05) level.

^a^ Children under 8 weeks old were not included in the analysis because data on maternal pertussis vaccination status were unavailable.

^b^ Adjusted for age, sex, ethnicity and deprivation.

^c^ African, Scottish African or British African, Caribbean or Black and other ethnic groups grouped into one category.

## Discussion

In this study, we demonstrated that childhood pertussis vaccination significantly reduced the odds of hospitalisation because of or with pertussis by 69% in all children aged 8 weeks to 17 years, and 88% in children aged 8 weeks to 1 year, after adjusting for confounding. We also illustrated the high incidence of potentially preventable hospitalisations due to pertussis, particularly in infants aged under 1 year. To our knowledge, this is the first study to quantify the protective effect of the childhood pertussis vaccination on hospitalisations caused by pertussis in Scotland. This provides valuable novel insights into the effectiveness of vaccination programmes in children in Scotland, and helps to understand the role of health inequalities.

Our data showed a protective effect of being fully vaccinated for age against hospital admissions with pertussis, consistent with the existing literature [10,11,17]. A study assessing vaccine effectiveness of pertussis-containing vaccine in children aged 0–14 years in Italy showed 88% statistically significant lower odds of hospital admissions in children vaccinated with a complete schedule [10]. Additionally, a study in France reported that odds of hospital admission with pertussis were 83% lower in children aged 1–2 years vaccinated with four doses of pertussis vaccine compared with unvaccinated children of the same age [17]. Additionally, studies assessing impact of maternal vaccination on preventing severe pertussis in newborns [18,19] report a significant protective impact on severity of disease.

We found that being partially vaccinated did not significantly reduce pertussis-associated hospital admissions in the multivariable model. The existing literature on this topic provides mixed findings. A study conducted in Italy involving children under 1 year of age reported results consistent with ours, showing no significant effect of receiving one or two doses of pertussis vaccine in preventing hospital admissions due to pertussis when compared with unvaccinated children (unadjusted OR: 1.56, p = 0.223) [10]. In contrast, a study from Sweden found a statistically significant protective effect of partial vaccination (one or two doses) against pertussis-associated hospital admissions in infants aged under 1 year [11].

Studies examining the effectiveness of pertussis-containing vaccines against disease incidence have demonstrated a significant protective effect of partial vaccination, although it is not as pronounced as being fully vaccinated [20,21]. A 2016 study by Crowcroft et al. reported statistically significant vaccine effectiveness against infection of 82.6% (95% CI: 72.3–89) and 76.7% (95% CI: 64.9–84.6) in partially vaccinated children within the age groups of 15–364 days and 1–3 years post-vaccination, respectively [21]. One possible explanation is that, while partial vaccination confers protection against pertussis infection, the level of protection may not be sufficient to prevent severe disease in the event of infection. However, our finding of no statistically significant protective effect of partial vaccination against severe disease in children with pertussis infection should be interpreted with caution. A larger study with increased statistical power may provide different results.

Our findings illustrate the importance of timely vaccination of children with each dose administered at the recommended age according to the childhood immunisations schedule. Vaccine coverage of the three primary doses of the 6-in-1 vaccine containing pertussis by children 12 months of age in Scotland was 94.4% in 2024 [6]. In addition to improving uptake and reducing inequalities, a key objective of a successful vaccination programme is to ensure timely and complete vaccination. Evidence from the UK shows that preventable hospital admissions in children have increased over time [22], and this increase has been attributed to a lack of preventative health interventions, including incomplete childhood immunisations [23]. This further contextualises the importance and novelty of our findings, with timely vaccinations not only preventing burden of ill-health on children and their families but also decreasing the burden of healthcare costs associated with these admissions.

Our finding that increasing age is associated with a reduced risk of pertussis-associated hospital admissions, with the highest number of admissions in infants under 1 year of age, is consistent with existing literature [10,24]. Additionally, studies have reported higher incidence rates of pertussis infections at population level in older children because of waning immunity [20,25-27]. While this was also seen in Scotland, a lower proportion of these cases resulted in hospital admissions, possibly due to the enhanced development of the immune system in older children.

Inequalities in childhood vaccination uptake by deprivation status have been documented in the UK, while children living in the most deprived areas have a higher incidence rate of pertussis infections [28]. The multivariable analysis in our study found that for children living in deprived areas, their vaccination status, age and ethnicity accounted for a significant proportion of the increased odds of hospital admissions with pertussis infections. This underscores the adverse impact of deprivation and its intersection with other demographic factors and determinants of health on the wellbeing of these children. Moreover, independent of the effect of vaccination status and other covariables, our findings also indicate that children living in the most deprived area, and those of Asian ethnicity, had increased odds of hospital admissions. This observation is consistent with data showing that individuals living in deprived areas and those belonging to ethnic minority groups are generally more likely to be admitted to hospital in the UK [29,30]. The underlying factors contributing to this higher rate including overall poorer health, higher obesity prevalence, and barriers to accessing preventative and primary care are beyond the scope of this study.

A strength and novel aspect of this study is the use of a Scotland level surveillance dataset, which was well characterised through deterministic linkage to other datasets at individual patient level. In addition, as pertussis is a legally notifiable disease in UK, the completeness of the data should be high. 

Our study had some limitations. We were unable to include children under 8 weeks of age in our analysis given the lack of individual maternal pertussis vaccination data at the time of the analysis. Although we adjusted for important confounders in our multivariable analysis, we were unable to adjust for other variables, such as premature birth, birthweight, breastfeeding and maternal vaccination status. We included Charlson Comorbidity Index as an indicator of children’s overall health status to address confounding bias. However, this index is only validated in adult populations and currently there are no standardised methods to assess multimorbidity in children [31]. We had 8% missing deprivation data, which we did include in the multivariable model to preserve statistical power. However, the effect size for this parameter showed a statistically significant increase in hospital admissions, indicating potential bias in our SIMD parameter estimates.

## Conclusion

This study demonstrates the significant protective effect of childhood pertussis vaccination in reducing hospitalisations from pertussis. These findings underscore the critical importance of vaccination programmes in protecting public health and reducing burden on healthcare systems. This study also highlights the need to address health inequalities by ensuring equitable access to vaccination services, especially in socioeconomically disadvantaged groups. Efforts to improve vaccine coverage, timeliness, and access, alongside addressing barriers to vaccination, are crucial in achieving optimal public health outcomes.

## Data Availability

The data supporting the findings of this study are not publicly available due to privacy and legal restrictions related to person identifiable health information. Access to anonymised data may be granted upon reasonable request to the relevant data protection authorities. Access is subject to their approval.

## Supplementary Data

117000 Supplement
