# Supplementary material for: Impact of vaccination on pertussis-related hospital admissions in children in Scotland from January 2013 to July 2024: a cohort study
Source: Euro Surveill. 2025 Oct 2;30(39):2500270. doi: 10.2807/1560-7917.ES.2025.30.39.2500270 (PMC12495382; doi:10.2807/1560-7917.ES.2025.30.39.2500270)
Supplement: Supplement [file 25-00270_HASAN_Supplement.pdf]

Supplementary materials:

"This supplementary material is hosted by Eurosurveillance as supporting information alongside the article [Impact of vaccination on pertussis-related hospital admissions in children in Scotland from January 2013 to July 2024: a cohort study], on behalf of the authors, who remain responsible for the accuracy and appropriateness of the content. The same standards for ethics, copyright, attributions and permissions as for the article apply. Supplements are not edited by Eurosurveillance and the journal is not responsible for the maintenance of any links or email addresses provided therein"

Supplementary table 1: Characteristics of the study population - children aged 17 and under with laboratory-confirmed pertussis in Scotland, by period of infection

Characteristics	2024			2013 - 2023		
	Total	Hospitalised n (%)	Non-hospitalised n (%)	Total	Hospitalised n (%)	Non-hospitalised n (%)
Case numbers	2665	84 (3.2%)	2581 (96.8%)	1317	180 (13.7%)	1137 (86.3%)
**Age groups**						
Under 8 weeks	26	19 (73.1%)	7 (26.9%)	48	39 (81.2%)	9 (18.8%)
Under 1s (excluding under 8 weeks)	128	40 (31.2%)	88 (68.8%)	172	88 (51.2%)	84 (48.8%)
1 – 4 years	319	11 (3.4%)	308 (96.6%)	216	24 (11.1%)	192 (88.9%)
5 – 9 years	696	3 (0.4%)	693 (99.6%)	211	12 (5.7%)	199 (94.3%)
10 – 14 years	1130	9 (0.8%)	1121 (99.2%)	457	15 (3.3%)	442 (96.7%)
15 – 17 years	366	2 (0.5%)	364 (99.5%)	213	2 (0.9%)	211 (99.1%)
**Sex**						
Females	1354	44 (3.2%)	1310 (96.8%)	705	90 (12.8%)	615 (87.2%)
Males	1311	40 (3.1%)	1271 (96.9%)	612	90 (14.7%)	522 (85.3%)
**Ethnicity**						
White	2382	72 (3%)	2310 (97%)	1212	163 (13.4%)	1049 (86.6%)
Asian	59	3 (5.1%)	56 (94.9%)	26	9 (34.6%)	17 (65.4%)
Black	4	1 (25%)	3 (75%)	0	0 (0)	0 (0)
Mixed	59	1 (1.7%)	58 (98.3%)	18	3 (16.7%)	15 (83.3%)
African	18	1 (5.6%)	17 (94.4%)	4	0 (0)	4 (100%)
Other	30	3 (10%)	27 (90%)	11	3 (27.3%)	8 (72.7%)
Missing	102	2 (2%)	100 (98%)	45	1 (2.2%)	44 (97.8%)
**Scottish Indices Multiple Deprivation (quintiles)**						
1st (most deprived)	489	33 (6.7%)	456 (93.3%)	211	51 (24.2%)	160 (75.8%)
2nd	458	17 (3.7%)	441 (95.3%)	179	30 (16.8%)	149 (83.2%)
3rd	499	15 (3%)	484 (97%)	182	22 (12.1%)	160 (87.9%)

4th	570	13 (2.3%)	557 (97.7%)	179	22 (12.3%)	157 (87.7%)
5th (least deprived)	644	6 (0.9%)	638 (99.1%)	245	19 (7.8%)	226 (92.2%)
Missing	5	0 (0%)	5 (100%)	321	36 (11.2%)	285 (88.8%)
**Vaccination status**						
Fully vaccinated for age	2250	32 (1.4%)	2218 (98.6%)	912	52 (5.7%)	860 (94.3%)
Partially vaccinated for age	151	10 (6.6%)	141 (93.4%)	171	26 (15.2%)	145 (84.8%)
Unvaccinated	264	42 (15.9%)	222 (84.1%)	234	102 (43.6%)	132 (56.4%)
**Comorbidities**						
No comorbidity	2567	79 (3.1%)	2488 (96.9%)	1233	175 (14.2%)	1058 (85.8%)
1-2 comorbidities	98	5 (5.1%)	93 (94.9%)	84	5 (6%)	79 (94%)

Percentages are presented by rows

[2] Each category is based on vaccination status, and time since last vaccine dose. As an example, category "Fully vaccinated for age-1 year" includes those pertussis cases that were fully vaccinated at the time of infection, and their last dose was within 365 before the date of infection

Supplementary table 2: Association between vaccination status and pertussis-associated hospital admissions in children aged 17 and under (excluding under 8 weeks old*) with laboratory confirmed pertussis infection in Scotland by period of onset

	Pertussis cases in 2024	Pertussis cases in 2013 - 2023
**Characteristic (n = 3908)[1]**	**Adjusted Odds Ratio (95% CI)[2]**	**Adjusted Odds Ratio (95% CI)[2]**
**Vaccination Status**		
Unvaccinated	reference	reference
Fully vaccinated for age	0.27(0.14-0.52) **	0.37(0.22-0.63) **
Partially vaccinated for age	0.98(0.38-2.4)	0.68(0.36-1.26)
**Age**		
Under 1 (excluding under 8 weeks)	reference	reference
1 - 3.5	0.11(0.05-0.24) **	0.30(0.16-0.56) **
3.6 - 9	0.02(0.01-0.05) **	0.08(0.04-0.15) **
10 - 12	0.02(0.01-0.06) **	0.07(0.03-0.15) **
13 - 17	0.04(0.01-0.08) **	0.03(0.01-0.06) **
**Sex**		
Female	Reference	reference
Male	0.55(0.3-0.97) *	1.11(0.72-1.69)
**Ethnicity**		
White	reference	reference
Asian	1.71(0.23-7.77)	2.93(0.82-9.5)
Other	0.69(0.18-2.07)	0.84(0.21-2.79)
Missing data	0.74(0.11-3.02)	0.87(0.12-3.34)
**Deprivation (SIMD Quintiles)**		
1 (most deprived)	1.81(0.69-5.38)	1.81(0.69-5.38)
2	1.41(0.49-4.44)	1.41(0.49-4.44)
3	1.63(0.56-5.13)	1.63(0.56-5.13)
4	1.57(0.53-4.97)	1.57(0.53-4.97)

5 (least deprived)	reference	reference
Missing data	NA	1.26(0.61-2.68)

[1]Children under 8 weeks old were removed from analysis due to unavailability of data on maternal pertussis vaccination status

[2]Adjusted for age, sex, ethnicity and deprivation

** p-value < 0.01

*p-value < 0.05
